# Effects of waste stream combinations from brewing industry on performance of Black Soldier Fly, *Hermetia illucens* (Diptera: Stratiomyidae)

**DOI:** 10.7717/peerj.5885

**Published:** 2018-11-28

**Authors:** Shaphan Y. Chia, Chrysantus M. Tanga, Isaac M. Osuga, Samira A. Mohamed, Fathiya M. Khamis, Daisy Salifu, Subramanian Sevgan, Komi K.M. Fiaboe, Saliou Niassy, Joop J.A. van Loon, Marcel Dicke, Sunday Ekesi

**Affiliations:** 1 Plant Health Theme, International Centre of Insect Physiology and Ecology (icipe), Nairobi, Kenya; 2 Laboratory of Entomology, Plant Sciences Group, Wageningen University, Wageningen, The Netherlands; 3 Department of Animal Sciences, Kenyatta University, Nairobi, Kenya

**Keywords:** *Hermetia illucens*, Protein quality, Net energy, Mass rearing, Quality control parameters, Agro-industrial by-products

## Abstract

**Background:**

In recent years, there has been a rapidly growing demand for readily accessible substrates for mass production of Black Soldier Fly, *Hermetia illucens* Linnaeus. Beer production results in various by-products that typically end up in uncontrolled dumpsites constituting pollution problems, which merits urgent attention. The present study investigated whether the 12 formulated diets composed of brewers’ spent grains (BSGs), brewers’ yeast and cane molasses can serve as substrate for *H. illucens* production.

**Methods:**

Four different BSGs were selected and formulated into 12 diets, aiming at varying protein and net energy levels. The diets were offered to newly hatched (∼1 h old) *H. illucens* larvae and the influence on developmental duration, survival, wet weight, pre-oviposition time, fecundity, and longevity were compared.

**Results:**

Developmental duration of the larvae (16–21 days) and pre-pupae (8–11 days) differed significantly across the different diets. The developmental duration of the pupae (8.7–9.1 days) was not affected by diet. The larval (86–99.2%), pre-pupal (71–95%), and pupal (65–91%) survival rates varied significantly between flies reared on the different diets. The pre-oviposition time was similar for flies provided with water (7–11 days) and 10% sugar solution (8–14 days) or across the different diets. The mean fecundity per female ranged from 324–787 eggs and did not differ between females provided with water or sugar solution. However, the number of eggs laid per female varied significantly across the different diets when provided with water. The longevity of starved *H. illucens* adults was significantly lower (5 days) compared to those provided with water (11–14 days) or sugar solution (14–15 days).

**Discussion:**

The implications of these findings as part of a quality control procedure for commercial production of high-quality *H. illucens* larvae as an alternative protein ingredient in livestock and aquaculture feed are discussed.

## Introduction

The United Nations figures project global human population growth of almost 50% since 2000 to 9.5 billion by 2050 (United Nations, Department of Economic and Social Affairs, Population Division, 2015). The increase in human population has resulted in an increase in the demand for protein and, consequently, an increase in the production of livestock, which is constrained by the availability of protein-rich feedstuffs (Tallentire, Mackenzie & Kyriazakis, 2018; Mottet et al., 2017; Herrero et al., 2015). Commonly used protein sources in livestock and aquaculture feeds include fish-derived and plant-derived protein sources, which are directly and indirectly competing with human nutrition (Van Der Spiegel, Noordam & Van Der Fels-Klerx, 2013; Shewry & Halford, 2002), creating an unsustainable pressure on the food value chain (Evans, 2009). Therefore, the development of innovative, cost-effective, and environmentally friendly options such as farming of insects on organic waste streams as alternative protein sources becomes important because they are increasingly considered an attractive, viable, and sustainable alternative to animal and plant protein sources (Henry et al., 2015; Makkar et al., 2014; Van Huis, 2013). Insects are rich in crude protein (35–77%), carbohydrate, fat, vitamins, and minerals (Henry et al., 2015; Makkar et al., 2014; Ganguly et al., 2013).

Insects like the Black Soldier Fly *Hermetia illucens* C. Linnaeus, offer promising alternatives of nutrient recovery while accumulating high-quality nutrient body biomass with an average of 42–43% crude protein, 33% fat and micronutrients such as iron and zinc (Barragán-Fonseca, 2018; Spranghers et al., 2017; Oonincx et al., 2015; Makkar et al., 2014; Rumpold & Schlüter, 2013). However, the nutritional status of these insects varies depending on the species and rearing substrates (Meneguz et al., 2018; Liland et al., 2017; Tschirner & Simon, 2015). The use of *H. illucens* larvae as an alternative to fishmeal or soybean meal in poultry, pig, and fish feeds has been advocated worldwide (Renna et al., 2017; Schiavone et al., 2017; Gasco et al., 2016; Ji et al., 2016; Lock, Arsiwalla & Waagbø, 2015; Veldkamp & Bosch, 2015; Makkar et al., 2014) and provides opportunities for income generation (Dobermann, Swift & Field, 2017; Kelemu et al., 2015; Van Huis et al., 2013). To meet with the increasing demand of high-quality *H. illucens*-based protein ingredients, mass production of *H. illucens* on readily available organic waste streams (Sanchez-Muros, Barroso & Manzano-Agugliaro, 2014) is important.

Organic waste management is a major challenge in Kenya, especially in Nairobi, the rapidly growing capital. In Nairobi, over 2,400 tons of waste are generated every day (Kasozi & Harro, 2010), of which only 38% is collected and less than 10% recycled (Japan International Cooperation Agency (JICA), 2010). The remaining 62% being organic waste largely from households, restaurants, hotels, markets, and agro-industrial manufacturing processes (Hoornweg & Bhada-Tata, 2012; UN-HABITAT, 2010a). For agro-industrial manufacturing processes in Kenya, Kenya Breweries Limited (KBL) and Mumias Sugar Company Limited generate huge amounts of waste. Only a small proportion of these massive waste streams has occasionally been used as supplements in livestock feed, since the advent of beer production in many countries in the world (Liguori et al., 2015; McDonald et al., 2002; Aliyu & Bala, 2011; Farhat et al., 2001; Calvert, 1991), but this is not the optimal use, as the spent grains are difficult for animals to digest (Newman & Jennings, 2008).

The use of *H. illucens* larvae to digest a wide range of organic waste streams, including animal manure (Xiao et al., 2018), fruit remains (Nguyen, Tomberlin & VanLaerhoven, 2013), and vegetable remains (Meneguz et al., 2018), or even some indigestible food such as coffee pulp (Diener, Zurbrügg & Tockner, 2009) has been well documented. Larvae of *H. illucens* can convert these organic waste streams to useful nutrients, maintaining a balance between high larval weight and reduction of organic solid matter up to about 42–56% (Nguyen, Tomberlin & Vanlaerhoven, 2015; Li et al., 2011; Diener, Zurbrügg & Tockner, 2009; Diener et al., 2011). Interest in the use of these waste streams as a source of value-added products is increasing rapidly due to their availability, year-round accessibility, affordability, low competitiveness for food or feed and the need for sustainable waste management procedures. According to Van Huis et al. (2013), Bioconversion of these waste streams using *H. illucens* will be more sustainable than other waste conversion and handling techniques as the insects are able to utilize massive amounts of organic waste and reduce the unpleasant smells emanating from the waste (Lardé, 1990), reduce efficiently the accumulation of polluting elements (nitrogen, phosphorus) from manure and compost (Beskin et al., 2018; Xiao et al., 2018; Sanchez-Muros, Barroso & Manzano-Agugliaro, 2014; Van Huis, 2013). Larvae of *H. illucens* also modify the microflora in organic waste thereby reducing the occurrence or abundance of undesirable bacteria (Yu et al., 2011; Erickson et al., 2004). Larvae of *H. illucens*, thus, add value to the waste (bio-fertilizers) and are efficient converters as they produce a protein and lipid-rich biomass from substrates that can be poorly used by monogastric animals (Xiao et al., 2018; Gobbi, Sánchez & Santos, 2013; Tomberlin & Sheppard, 2002; Tomberlin, Sheppard & Joyce, 2002). These characteristics, linked to a short production cycle, make *H. illucens* larvae very good candidates for intensive production. Therefore, waste that would otherwise contaminate the environment and put human and animal health at risk could be a source of income generation and employment creation through well-established recycling and resource recovery (Liguori et al., 2015; Nguyen, Tomberlin & VanLaerhoven, 2013; Diener, Zurbrügg & Tockner, 2009; Diener et al., 2011; UN-HABITAT, 2010b).

Although, the economic importance of this fly as a potential candidate for mass rearing is well established, knowledge on important aspects of the reproductive biology of *H. illucens* on agro-industrial waste streams (mixed diets of brewer’s spent grains (BSGs), brewers’ yeast, and cane molasses) as suitable substrates for mass production remains largely unknown. The process of beer and sugar manufacturing generates various by-products, typically BSGs, brewers’ yeast, and molasses. These by-products are produced in large quantities daily, readily available and highly accessible throughout the year and easy to handle. Here, we investigate the suitability of these waste streams as substrate for *H. illucens*.

It is well known that the quality of larval diet significantly affects mass rearing of insects, especially growth, survival, and biological traits of adult flies because large females have large ovaries and lay more eggs than small females (Gobbi, Sánchez & Santos, 2013; Blackmore & Lord, 2000; Roper, Pignatelli & Partridge, 1996; Churchill-Stanland et al., 1986). Thus, larval diet quality and feeding are crucial to overall fitness (Moreau, Benrey & Thiéry, 2006; Tomberlin, Sheppard & Joyce, 2002; Tikkanen, Niemela & Keranen, 2000). In this study, we combined different agro-industrial waste streams and determined the life-history parameters of *H. illucens* by focusing on the following research questions: how does the quality of the larval diet affect (a) developmental duration of immature life stages, (b) their survival, (c) larval, pre-pupal, pupal, and adult biomass, (d) pre-oviposition duration, (e) adult fecundity, and (f) longevity of starved, water-provided and sugar-fed adults.

## Materials and Methods

### Insect culture

This study was carried out at the Animal Rearing and Containment Unit of the International Centre of Insect Physiology and Ecology (*icipe*), Nairobi, Kenya. *H. illucens* colony was established in 2016 from eggs of wild-trapped *H. illucens* populations in Kasarani, Nairobi County (S 01°13′14.6″; E 036°53′44.5″, 1,612 m a.s.l.) following the method described by Sripontan, Juntavimon & Chiu (2017) and Booth & Sheppard (1984) with slight modifications. The egg clusters were transferred to metal trays (76 × 27.5 × 10 cm) containing 2,000 g of BSG diluted in 3,200 ml of water. The diet was hydrated to approximately 70 ± 2% moisture by weight and confirmed using a moisture sensor with two 12 cm long probes (HydroSense™ CS620; Campbell Scientific, Inc., Logan, UT, USA). The culture was monitored daily for larval development. The pre-pupal stages after self-dispersal from the substrate were kept in four l transparent rectangular plastic containers (21 × 14 × 15 cm) (Kenpoly Manufacturer Ltd., Nairobi, Kenya) containing moist wood shavings (sawdust) as pupation substrate according to Holmes, Vanlaerhoven & Tomberlin (2013). An opening (14.5 × 8.3 cm) was made in the lid of each container and covered with fine netting organza material capable of retaining emerging adult flies. Conditions in the rearing room were maintained at 28 ± 1 °C, 70 ± 2% relative humidity (RH) and a photoperiod of L12:D12.

Adults were transferred to outdoor cages (1 × 1 × 1.8 m) where water and sugar solution were provided ad libitum to the flies. When the adult flies in the cage were 7-days-old (Nakamura et al., 2016), moist chicken manure (500 g diluted in 800 ml of water) was provided in plastic containers (30 × 15 cm) with the surface covered with wire mesh. Strips of cardboard with flutes along the edges were placed on top of the wire mesh, which provided the flies with sites for laying eggs. The containers were checked daily to collect egg clusters deposited by the flies. The cardboard strips with egg clusters were transferred to plastic containers and placed in climate-controlled chambers. The newly hatched larvae were fed BSG ad libitum until full development into pre-pupal stages. The pre-pupal stages were transferred into two l transparent rectangular plastic containers containing a 2.5 cm layer of moist wood shavings and monitored daily for pupal formation. The pupae collected were regularly transferred into four l transparent plastic rectangular containers containing a 2.5 cm layer of moist wood shavings until emergence. The emerged flies were transferred to the outdoor rearing cages designed specifically to hold the adult fly stock populations. The *H. illucens* colony has been in culture for ∼2 years and once every 6 months, wild-caught flies are added to the colony to prevent inbreeding depression. In addition, adult *H. illucens* populations in the cages were maintained in low numbers (approximately 2,000 adult flies in a 1 × 1.2 × 1.8 m cage) to avoid stressful crowding effects, which is very common in insect mass production (Sørensen & Loeschcke, 2001).

### Experimental substrates and diet formulation

The BSGs used were sourced once from the KBL, Nairobi, Kenya; main producer of major beer brands in the country: Tusker (malt and corn starch); Guinness (malt and barley); Senator (sorghum and barley), and Pilsner (barley). The liquid form of brewer’s yeast from the processing of each of the beer brands was also collected as part of waste streams to be used during the experiments. The fresh BSGs were placed on plastic sheets with moving dry air (28.0 ± 1 °C) at ambient temperature for 48 h using an Xpelair^®^ heater (WH30, 3 KW Wall Fan Heater; Peterborough, UK). Possible fermentation of the BSGs at this temperature was avoided by turning the substrates twice daily to ensure proper aeration and to prevent molding within the substrates. Thereafter, the semi-dried products were oven-dried at 60 °C for 72 h to approximately 90% dry matter (DM) (∼10% moisture). The dried BSGs were later passed through a three mm sieve in a Münch hammer mill (Münch, Wuppertal, Germany) to obtain particle size suitable for incorporation into *H. illucens* diet. Molasses was obtained in liquid form from Mumias Sugar Company Limited.

Dried BSGs from the four main beer brands were each provided with three treatments to obtain 12 different diets as follows: The first group was the “control” for which 50 g of each BSG was mixed with 80 ml of water only: malt/corn-starch/water; malt/barley/water; sorghum/barley/water, and barley/water. Each diet was hydrated to approximately 70 ± 2% moisture by weight and confirmed using a moisture sensor with two 12 cm long probes (HydroSense™ CS620; Campbell Scientific, Inc., Logan, UT, USA). In the second group, each of four BSGs was supplemented with waste brewer’s yeast. Fifty grams of each BSG was mixed with 90 ml of brewer’s yeast to generate the following treatments: malt/corn-starch/brewer’s yeast; malt/barley/brewer’s yeast; sorghum/barley/brewer’s yeast, and barley/brewer’s yeast. In the third group, 50 g of each BSG was supplemented with 45 ml of waste brewers’ yeast + 45 ml of molasses: malt/corn-starch/brewer’s yeast/molasses; malt/barley/brewer’s yeast/molasses; sorghum/barley/brewer’s yeast/molasses, and barley/brewer’s yeast/molasses.

### Chemical analysis of experimental diets

Prior to conducting proximate analysis of the various diets using the method described by Association of Official Analytical Chemists (1990), weighed samples were oven-dried at 60 °C for 72 h. DM content of each sample was measured by oven drying at 105 °C for 48 h until constant weight was achieved (Pen et al., 2013; Association of Official Analytical Chemists, 1990). Moisture content was determined using the oven set at 105 °C for 24 h (Okedi, 1992; Association of Official Analytical Chemists, 1990). Nitrogen content was determined using the Kjeldahl method (Association of Official Analytical Chemists, 1990) and later converted to crude protein content by multiplying with factor 6.25 (Finke, 2007). The ash content was determined using a muffle furnace and samples heated at 550 °C overnight according to the method described by Association of Official Analytical Chemists (1990). Velp solvent extractor (SER 148/6) was used to determine fat content (crude fat) with ethyl ether as extractant (Association of Official Analytical Chemists, 1990). Total carbohydrate was calculated by difference using the standard methods (Association of Official Analytical Chemists, 1990; Kirk, Sawyer & Pearson, 1981). All parameters discussed above were determined in triplicate per sample and expressed as a percentage.

The net energy (NE) value of the various experimental diets was measured by indirect calorimetry (Li et al., 2015, 2018; Velayudhan, Heo & Nyachoti, 2015; Liu et al., 2014, 2015; Heo, Adewole & Nyachoti, 2014). The NE value of feedstuffs reflects the true availability of energy to the insect and remains the most accurate and unbiased way to date of characterizing the energy content of feed (Moehn, Atakora & Ball, 2005).

### Experimental design

Before the start of the experiment, the rearing room was maintained at 28.0 ± 1 °C using an Xpelair^®^ heater (WH30, 3 KW Wall Fan Heater; Peterborough, UK). The RH in the experimental room was adjusted and maintained at 70 ± 2% using an adiabatic atomizer humidifier (Condair ABS3; Hornsby, Australia), while maintaining 12:12 L:D photoperiod. The condition of the room was monitored daily using a WiFi Sensor (WiFi-TH Corintech Ltd., Fordingbridge, UK; Firmware version 5.1.7/13.3.3G/R4.11). Thereafter, 120 egg batches (∼3 h old) collected from the adult stock culture maintained in the outdoor cages described above were distributed equally in 12 sterilized disposable 100 × 15 mm Petri dishes and monitored at 6 h intervals daily for egg eclosion.

According to the method described by Gobbi, Sánchez & Santos (2013), 300 neonate larvae (∼1 h old) were individually counted with the aid of entomological tweezers and a moist fine camel hair brush under a stereomicroscope (Leica MZ 125 Microscope; Leica Microsystems Switzerland Limited, Heerbrugg, Switzerland), fitted with a Toshiba 3CCD camera using the Auto-Montage software (Syncroscopy; Synoptics Group, Cambridge, UK) at magnification of ×25. The larvae were carefully lined on moistened pieces of sterilized black cloth, which were thereafter placed on top of the experimental diet in each of the 12 transparent plastic containers (12 × 4.5 cm). The lid of each container was designed with an opening (8 × 4 cm) fitted with fine netting material of 1.3 × 1.3 mm mesh size for ventilation. Each experimental setup was then maintained in the climate-controlled rearing room described above. The larvae in each treatment were provided ample feeding substrate to carry them throughout the larval developmental phase to prepupae (non-feeding phase). The larvae generally have a cream-like color but at the fifth instar stage there is a recognizable on-set of exoskeletons (skin) color change to beige (dark brown) before they undergo the last molt to the charcoal-grey colored prepupal stage (Dortmans et al., 2017). Once the larvae turned into pre-pupae, they were transferred individually into plastic containers (3 × 4 × 3 cm) with 2.5 cm layer of moist wood shavings (sawdust). Each container had an opening (2.5 cm diameter) covered with fine netting organza material for ventilation. The containers were checked daily, and pupae formed were recorded. The pupae were collected and maintained individually in similar plastic containers until emergence. Stage-specific developmental time, survival and wet weight were calculated for each treatment. Weight measurements of the different life stages were carried out using a Kern-PCB 350-3 precision balance (0.001–350 g). The experiments were replicated five times for each experimental diet.

### Pre-oviposition period, oviposition period, fecundity, and longevity when starved or provided with water or sugar solution

To determine the effect of each of the 12 experimental larval diets on life-history parameters, ninety paired newly emerged (<24 h old) adult flies were randomly selected by collecting fully winged male and female flies that emerged from each dietary treatment. The paired adult flies were subdivided into three groups of 30 each. Individual pairs of flies from each group were kept in transparent rectangular Perspex cages (30 × 16 × 16 cm) with openings covered with breathable material. Two strips of cardboard with holes along the edges were provided for laying eggs. The first group of paired flies from each diet were starved (unfed) throughout the experiment, while the second and third groups were provided with water and 10% sugar solution on soaked cotton wool, respectively. Each experimental set-up was observed daily to record the number of eggs laid. The pre-oviposition period was calculated from the first day of emergence of an adult female to the first day of oviposition. Eggs laid on each day were collected with the aid of a fine wet black camel hair brush. Each egg clutch collected was physically separated by spreading it on the surface of an electrically powered light box (2 × 15 W 6,500 K, model 44077 B.S.4533; Sasco, London, England) and counted with the help of a tally counter. The light box allowed for easy identification of individual eggs during counting. The experiment was terminated when the female and male flies died. Both longevity and fecundity were calculated for each diet.

### Statistical analysis

Larval weight, pre-pupal weight, pupal weight, adult weight, development duration, adult longevity, number of eggs (female fecundity), and pre-oviposition period were subjected to analysis of variance (ANOVA) to evaluate the effect of the waste streams on these variables. Number of eggs was log-transformed prior to ANOVA to stabilize variance. Tukey’s Honestly Significant Difference test was used to separate means. A *t*-test was used to compare the pre-oviposition period between treatments with sugar solution and water within each experimental diet. Data was summarized and presented as means ± standard error (SE). Further, orthogonal contrasts were created and evaluated using the glht function in the multcomp package (Hothorn, Bretz & Westfall, 2008) to explore the structure in the treatments, the agro-industrials wastes. The differences among treatment means were considered statistically significant at α = 0.05. All statistical analyses were implemented using R version 3.3.3 (R Core Team, 2017).

## Results

### Nutrient composition of experimental diets

Marked variation was observed on the nutritional composition (on DM basis) of the diets used in this study (Table 1). There was a significant difference in crude protein (*F* = 194.90; d*f* = 11, 24; *P* < 0.0001), crude fat (*F* = 45.09; d*f* = 11, 24; *P* < 0.0001), ash (*F* = 8.48; d*f* = 11, 24; *P* < 0.0001), moisture (*F* = 261.90; d*f* = 11, 24; *P* < 0.0001), total carbohydrate (*F* = 17.05; d*f* = 11, 24; *P* < 0.0001), and NE (*F* = 39.97; d*f* = 11, 24; *P* < 0.0001) contents among the experimental diets. Only diets supplemented with brewers’ yeast had higher crude protein levels compared to the other diets. The inclusion of brewers’ yeast and molasses in diets (spent grains) resulted in lower crude protein contents compared to diets mixed with water only. The NE values were equally higher for diets supplemented with brewers’ yeast (Table 1).

**Table 1:** Nutrient composition (on dry matter basis) of experimental diets.

Larval diet	Crude protein (%)	Crude fat (%)	Ash (%)	Moisture (%)	Net energy (Kcal/g)
BW	30.33 ± 0.24^bc^	6.38 ± 0.17^c^	4.15 ± 0.18^bcd^	11.57 ± 0.10^cd^	3.44 ± 0.02^ef^
MBW	28.89 ± 0.23^cd^	6.78 ± 0.25^c^	3.80 ± 0.19^cd^	12.38 ± 0.13^c^	3.77 ± 0.02^cd^
MCW	27.38 ± 0.11^d^	6.46 ± 0.41^c^	3.03 ± 0.18^d^	12.10 ± 0.15^c^	3.66 ± 0.01^cde^
SBW	29.43 ± 0.30^c^	11.8 ± 1.07^a^	3.72 ± 0.18^cd^	10.38 ± 0.13^def^	3.85 ± 0.03^c^
BY	31.99 ± 0.08^a^	5.39 ± 0.03^cd^	4.74 ± 0.28^abc^	11.53 ± 0.12^cd^	4.16 ± 0.09^a^
MBY	30.22 ± 0.10^bc^	6.96 ± 0.13^c^	4.41 ± 0.34^bc^	10.76 ± 0.26^de^	4.11 ± 0.06^ab^
MCY	27.72 ± 0.23^d^	6.04 ± 0.15^c^	5.14 ± 0.2^ab^	9.91 ± 0.11^ef^	4.22 ± 0.05^a^
SBY	31.39 ± 0.17^ab^	9.48 ± 0.15^b^	4.32 ± 0.25^bc^	9.17 ± 0.20^f^	3.87 ± 0.06^bc^
BYMo	22.14 ± 0.48^e^	3.95 ± 0.31^def^	5.8 ± 0.19^a^	19.51 ± 0.23^a^	3.56 ± 0.03^de^
MBYMo	22.32 ± 0.71^e^	3.23 ± 0.17^f^	4.08 ± 0.22^bcd^	18.24 ± 0.20^b^	3.65 ± 0.05^cde^
MCYMo	19.10 ± 0.35^f^	3.42 ± 0.02^ef^	4.31 ± 0.21^bc^	18.79 ± 0.62^ab^	3.44 ± 0.05^ef^
SBYMo	21.69 ± 0.14^e^	5.18 ± 0.15^cde^	4.71 ± 0.20^abc^	18.66 ± 0.22^ab^	3.29 ± 0.05^f^

**Notes:**

Means in a column followed by different lowercase letter are significantly different (*P* < 0.05, ANOVA plus HSD).

BW, Barley/water; MBW, Malt/Barley/water; MCW, Malt/Corn-starch/water; SBW, Sorghum/Barley/water; BY, Barley/brewer’s yeast; MBY, Malt/Barley/brewer’s yeast; MCY, Malt/Corn-starch/brewer’s yeast; SBY, Sorghum/Barley/brewer’s yeast; BYMo, Barley/brewer’s yeast/Molasses; MBYMo, Malt/Barley/brewer’s yeast/Molasses; MCYMo, Malt/Corn-starch/brewer’s yeast/Molasses and SBYMo, Sorghum/Barley/Molasses.

### Effect of rearing diet on development of immature stages of *H. illucens*

There were significant differences in larval (*F* = 14.16; d*f* = 11, 48; *P* < 0.001) and pre-pupal (*F* = 12.45; d*f* = 11, 48; *P* < 0.001) developmental time among experimental diets (Table 2). Pupal development time did not differ significantly between diets (*F* = 0.89; d*f* = 11, 48; *P* = 0.55). Total developmental time (larva-adult) was significantly different among diets (*F* = 40.57; d*f* = 11, 96; *P* < 0.001). Development time was similar for males and females from the same diet (Table 2) and there was no significant interaction (*F* = 0.09; d*f* = 11, 96; *P* = 1.0) between diet and sex. Larval developmental time did not differ significantly between non-supplemented diet vs. diets supplemented with brewers’ yeast only.

**Table 2:** Development time (day ± SE) of *H. illucens* stages and comparison between treatment (diet) groups using orthogonal contrasts.

Diet	Larva (days)	Pre-pupa (days)	Pupa (days)	Adult (Larval-adult)	
				Male (days)	Female (days)
BW	17.2 ± 0.5^cde^	8.8 ± 0.1^bcd^	9.1 ± 0.1^a^	35.0 ± 0.5^cdA^	35.2 ± 0.5^cdA^
MBW	16.4 ± 0.2^e^	9.5 ± 0.1^abc^	8.7 ± 0.2^a^	34.5 ± 0.4^dA^	34.7 ± 0.3^dA^
MCW	18.3 ± 0.4^bcd^	8.3 ± 0.1^cd^	8.8 ± 0.1^a^	35.5 ± 0.6^cdA^	35.5 ± 0.6^cdA^
SBW	20.6 ± 0.3^a^	8.8 ± 0.3^bcd^	9.0 ± 0.0^a^	38.3 ± 0.5^abA^	38.4 ± 0.4^abA^
BY	17.9 ± 0.5^bcde^	10.2 ± 0.3^a^	8.9 ± 0.1^a^	37.0 ± 0.3^bcA^	37.0 ± 0.3^bcA^
MBY	19.1 ± 0.4^ab^	10.3 ± 0.3^a^	8.8 ± 0.1^a^	38.2 ± 0.6^abA^	38.3 ± 0.4^abA^
MCY	19.0 ± 0.3^ab^	10.5 ± 0.4^a^	9.0 ± 0.1^a^	38.5 ± 0.7^abA^	38.5 ± 0.5^abA^
SBY	16.6 ± 0.4^de^	8.5 ± 0.2^cd^	8.8 ± 0.1^a^	34.1 ± 0.4^dA^	33.7 ± 0.4^dA^
BYMo	19.4 ± 0.3^ab^	10.9 ± 0.4^a^	9.0 ± 0.1^a^	39.3 ± 0.4^aA^	39.3 ± 0.5^aA^
MBYMo	19.5 ± 0.3^ab^	10.0 ± 0.3^ab^	8.8 ± 0.1^a^	38.3 ± 0.4^abA^	38.2 ± 0.4^abA^
MCYMo	20.2 ± 0.2^a^	10.4 ± 0.4^a^	9.0 ± 0.2^a^	39.4 ± 0.3^aA^	39.8 ± 0.2^aA^
SBYMo	18.5 ± 0.2^bc^	8.1 ± 0.1^d^	8.9 ± 0.1^a^	35.4 ± 0.3^cdA^	35.4 ± 0.2^cdA^

Contrasts					
	d*f* = 1, 48	d*f* = 1, 48		d*f* = 1, 48	d*f* = 1, 48
Non-supplemented vs. Yeast/molasses + yeast supplemented diets	*F* = 8.768, *P* = **0.044**	*F* = 33.919, *P* < **0.0001**	ns	*F* = 35.83, *P* < **0.0001**	*F* = 38.53, *P* < **0.0001**
Non-supplemented vs. Yeast only	*F* = 0.008, *P* = 1.000	*F* = 27.974, *P* < **0.0001**	ns	*F* = 11.83, *P* = **0.012**	*F* = 10.06, *P* = **0.025**
Yeast vs. molasses/yeast	*F* = 24.552, *P* < **0.0001**	*F* = 0.241, *P* = 1.000	ns	*F* = 12.17, *P* = **0.010**	*F* = 19.42, *P* < **0.0001**
Barley vs. Corn-starch	*F* = 12.624, *P* = **0.008**	*F* = 0.762, *P* = 0.98	ns	*F* = 3.24, *P* = 0.49	*F* = 5.11, *P* = 0.22
Barley vs. Sorghum	*F* = 1.836, *P* = 0.80	*F* = 44.449, *P* < **0.0001**	ns	*F* = 9.97, *P* = **0.026**	*F* = 15.77, *P* = **0.003**

**Notes:**

Means in a column followed by the same lowercase letter are not significantly different (*P* < 0.05, ANOVA plus HSD). Means for both sexes within a treatment followed by the same uppercase letter are not significantly different.

*P*-values in bold indicate significant difference; ns, not significantly different (*P* < 0.05).

BW, Barley/water; MBW, Malt/Barley/water; MCW, Malt/Corn-starch/water; SBW, Sorghum/Barley/water; BY, Barley/brewer’s yeast; MBY, Malt/Barley/brewer’s yeast; MCY, Malt/Corn-starch/brewer’s yeast; SBY, Sorghum/Barley/brewer’s yeast; BYMo, Barley/brewer’s yeast/Molasses; MBYMo, Malt/Barley/brewer’s yeast/Molasses; MCYMo, Malt/Corn-starch/brewer’s yeast/Molasses and SBYMo, Sorghum/Barley/Molasses. Non-supplemented diets = BW, MBW, MCW, and SBW; Yeast/Molasses + yeast-supplemented diets = BY, MBY, MCY, SBY, BYMo, MBYMo, MCYMo, and SBYMo; Yeast-based diets = BY, MBY, MCY, and SBY; Molasses-based diets = BYMo, MBYMo, MCYMo, and SBYMo; Barley-based diets = BW, BY, and BYMo; Corn-starch-based diets = MCW, MCY, and MCYMo; Sorghum-based diets = SBW, SBY, and SBYMo.

### Effect of diet on the larval survival, pre-pupal survival, and pupal survival of *H. illucens*

**Figure 1:**
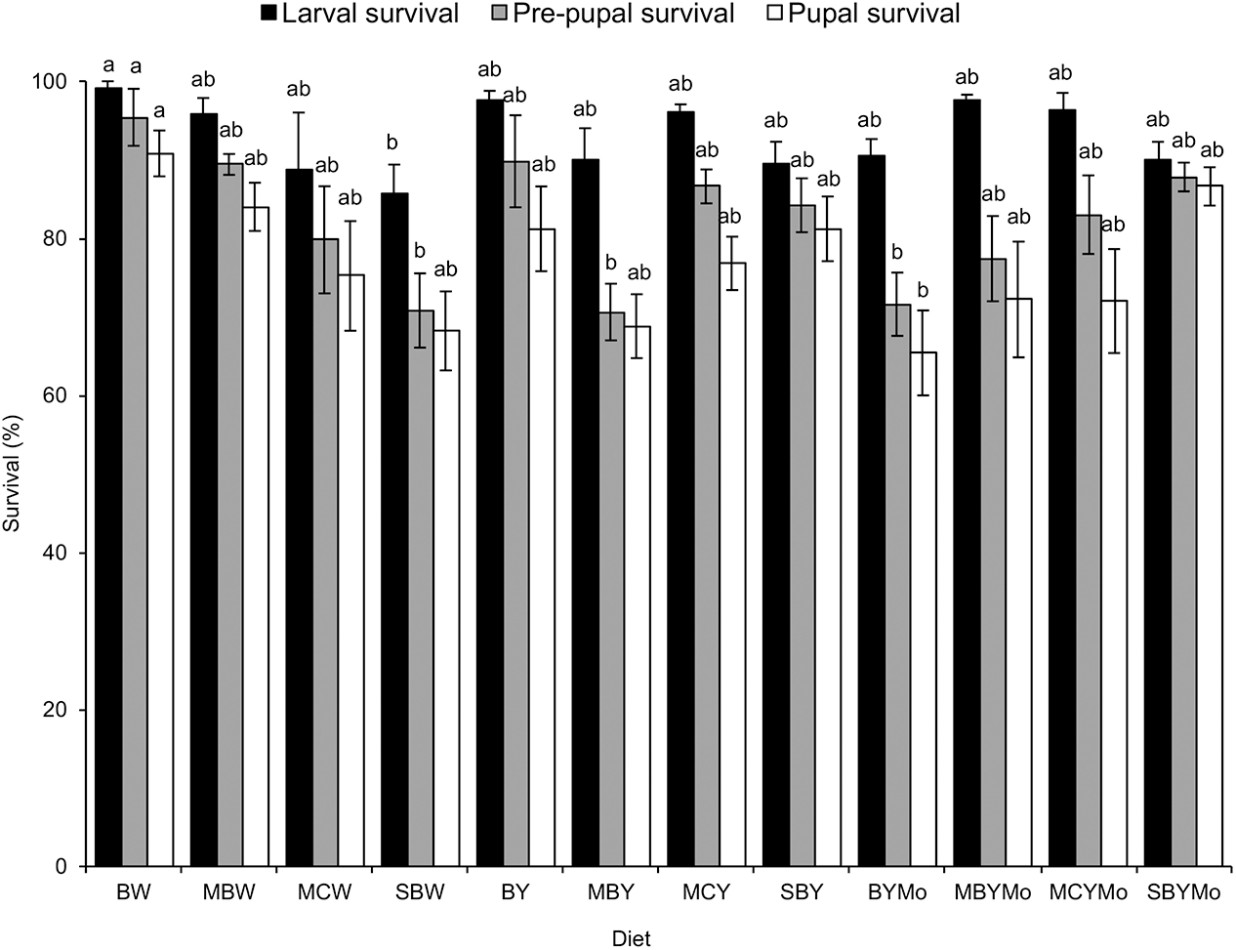
Stage-specific survival of *H. illucens* reared on various larvae diets. Means (±SE) followed by the same lowercase letter for a given life stage are not significantly different (*P* < 0.05, ANOVA followed by HSD). BW, Barley/water; MBW, Malt/Barley/water; MCW, Malt/Corn-starch/water; SBW, Sorghum/Barley/water; BY, Barley/brewer’s yeast; MBY, Malt/Barley/brewer’s yeast; MCY, Malt/Corn-starch/brewer’s yeast; SBY, Sorghum/Barley/brewer’s yeast; BYMo, Barley/brewer’s yeast/Molasses; MBYMo, Malt/Barley/brewer’s yeast/Molasses; MCYMo, Malt/Corn-starch/brewer’s yeast/Molasses and SBYMo, Sorghum/Barley/Molasses.

Larval survival (*F* = 2.13; d*f* = 11, 48; *P* = 0.036), pre-pupal survival (*F* = 3.67; d*f* = 11, 48; *P* = 0.001), and pupal survival (*F* = 2.54; d*f* = 11, 48; *P* = 0.013) were significantly affected by diet type (Fig. 1). Orthogonal contrasts between treatment groups showed no significant differences in larval survival, pre-pupal survival, and pupal survival between diets supplemented with brewers’ yeast and the non-supplemented diets (Table 3).

**Table 3:** Comparison of stage-specific survival between treatment groups using orthogonal contrasts for the *H. illucens*.

Contrast	Larva	Pre-pupa	Pupa
	d*f* = 1, 48	d*f* = 1, 48	d*f* = 1, 48
Non-supplemented vs. Yeast/Molasses + yeast-supplemented diets	*F* = 0.58, *P* = 0.99	*F* = 0.88, *P* = 0.97	*F* = 1.73, *P* = 0.82
Non-supplemented vs. Yeast	*F* = 0.36, *P* = 1.0	*F* = 0.12, *P* = 1.0	*F* = 0.52, *P* = 0.99
Yeast vs. molasses + yeast	*F* = 0.01, *P* = 1.0	*F* = 0.88, *P* = 0.97	*F* = 0.70, *P* = 0.99
Barley vs. Corn-starch	*F* = 0.69, *P* = 0.99	*F* = 0.49, *P* = 1.0	*F* = 1.20, *P* = 0.92
Barley vs. Sorghum	*F* = 7.46, *P* = 0.078	*F* = 1.77, *P* = 0.81	*F* = 0.01, *P* = 1.0

**Note:**

Non-supplemented diets = BW, MBW, MCW, and SBW; Yeast/Molasses + yeast-supplemented diets = BY, MBY, MCY, SBY, BYMo, MBYMo, MCYMo, and SBYMo; Yeast-based diets = BY, MBY, MCY, and SBY; Molasses/yeast diets = BYMo, MBYMo, MCYMo, and SBYMo; Barley-based diets = BW, BY, and BYMo; Corn-starch = MCW, MCY, and MCYMo; Sorghum-based diets = SBW, SBY, and SBYMo.

### Effect of larval diet on pre-oviposition period, fecundity, and longevity of Black Soldier Fly

Pre-oviposition time of adult *H. illucens* provided with a 10% sugar solution (*F* = 2.36; d*f* = 1, 75; *P* = 0.08) or water (*F* = 0.38; d*f* = 1, 75; *P* = 0.46) was not significantly affected by larval diet (Table 4). On all diets, starved adult female flies failed to reach oviposition age as the female could only survive for a maximum of 6 days.

**Table 4:** Pre-oviposition period (mean ± SE) of female *H. illucens* fed on sugar solution and water.

Larval diet	Pre-oviposition period (days)	
	Sugar solution	Water
BW	9.5 ± 0.5^aA^	9.2 ± 1.1^aA^
MBW	8.8 ± 0.5^aA^	9.6 ± 0.9^aA^
MCW	10.0 ± 1.5^aA^	11.0 ± 2.0^aA^
SBW	8.0 ± 0.0^aA^	7.3 ± 0.8^aA^
BY	13.5 ± 3.5^aA^	8.2 ± 0.7^aB^
MBY	9.8 ± 0.3^aA^	10.2 ± 2.4^aA^
MCY	9.0 ± 0.6^aA^	7.8 ± 0.5^aA^
BYMo	10.5 ± 0.5^aA^	7.5 ± 0.5^aA^
MBYMo	10.2 ± 1.1^aA^	8.0 ± 1.1^aA^
MCYMo	10.2 ± 1.0^aA^	8.4 ± 0.4^aA^
SBYMo	10.7 ± 3.7^aA^	7.0 ± 0.3^aA^

**Note:**

Means in a column followed by the same lowercase letter are not significantly different (*P* < 0.05, HSD). Means for both sugar and water-fed female BSF within each treatment followed by the same uppercase letter are not significantly different (*P* < 0.05, *t*-test).

**Figure 2:**
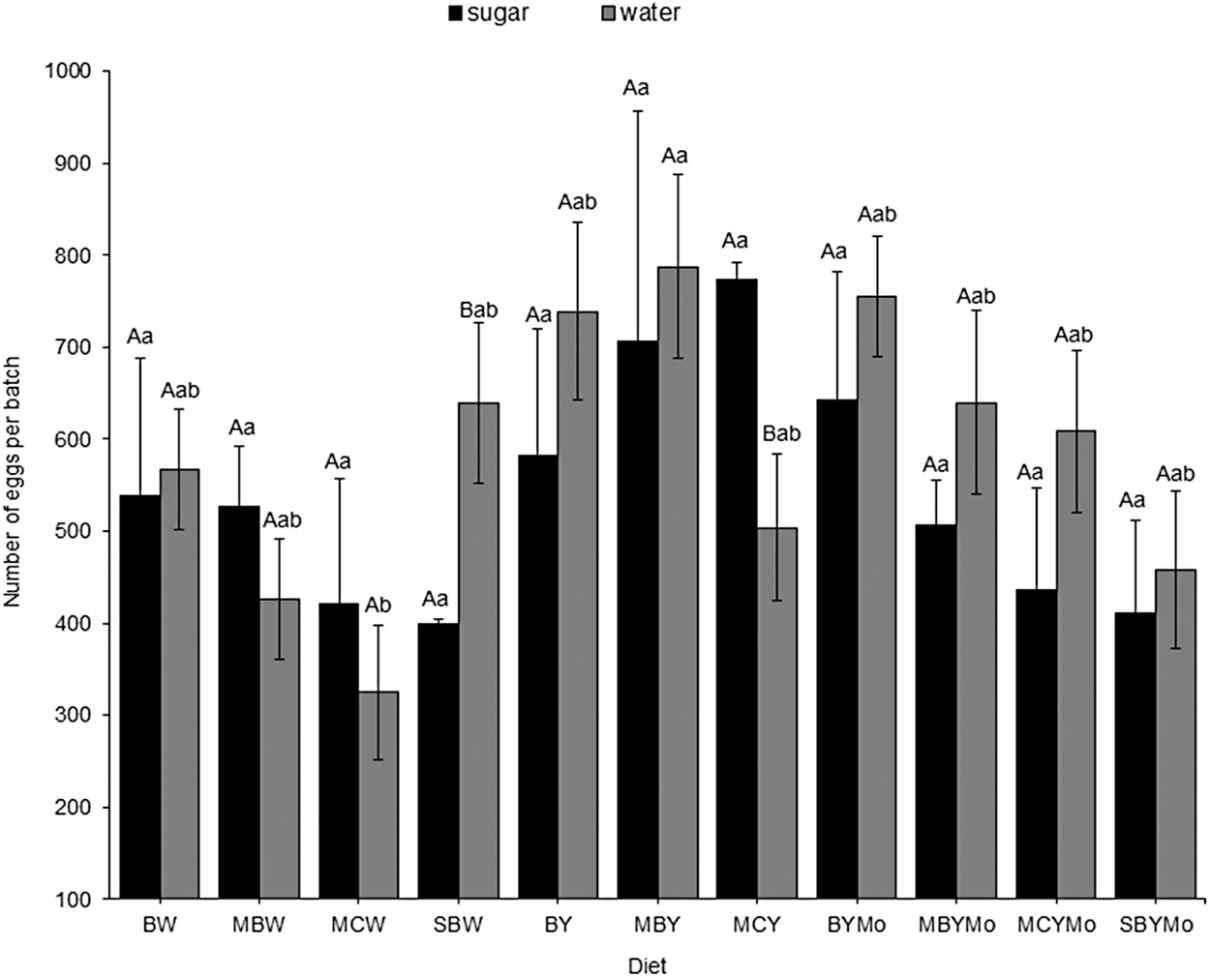
Mean number of eggs laid per adult female *H. illucens* reared on different larval diets. Means (±SE) followed by different upper-case letter are significantly different between sugar- and water-fed flies for each diet. Means followed by different lower-case letter are significantly different among diets (*P* < 0.05, HSD). BW, Barley/water; MBW, Malt/Barley/water; MCW, Malt/Corn-starch/water; SBW, Sorghum/Barley/water; BY, Barley/brewer’s yeast; MBY, Malt/Barley/brewer’s yeast; MCY, Malt/Corn-starch/brewer’s yeast; BYMo, Barley/brewer’s yeast/Molasses; MBYMo, Malt/Barley/brewer’s yeast/Molasses; MCYMo, Malt/Corn-starch/brewer’s yeast/Molasses and SBYMo, Sorghum/Barley/Molasses.

The fecundity of female flies provided with sugar solution was similar (*F* = 0.73; d*f* = 10, 27; *P* = 0.69) across dietary treatments but varied significantly from diets provided with water (*F* = 2.89; d*f* = 10, 38; *P* = 0.010) (Fig. 2). The fecundity of female flies was higher for almost all the diets supplemented with brewers’ yeast or molasses/brewers’ yeast than for the non-supplemented diets (Fig. 2). Egg production was similar (*F* = 0.88; d*f* = 1, 85; *P* = 0.35) for female flies provided with water or sugar solution (Fig. 2).

**Figure 3:**
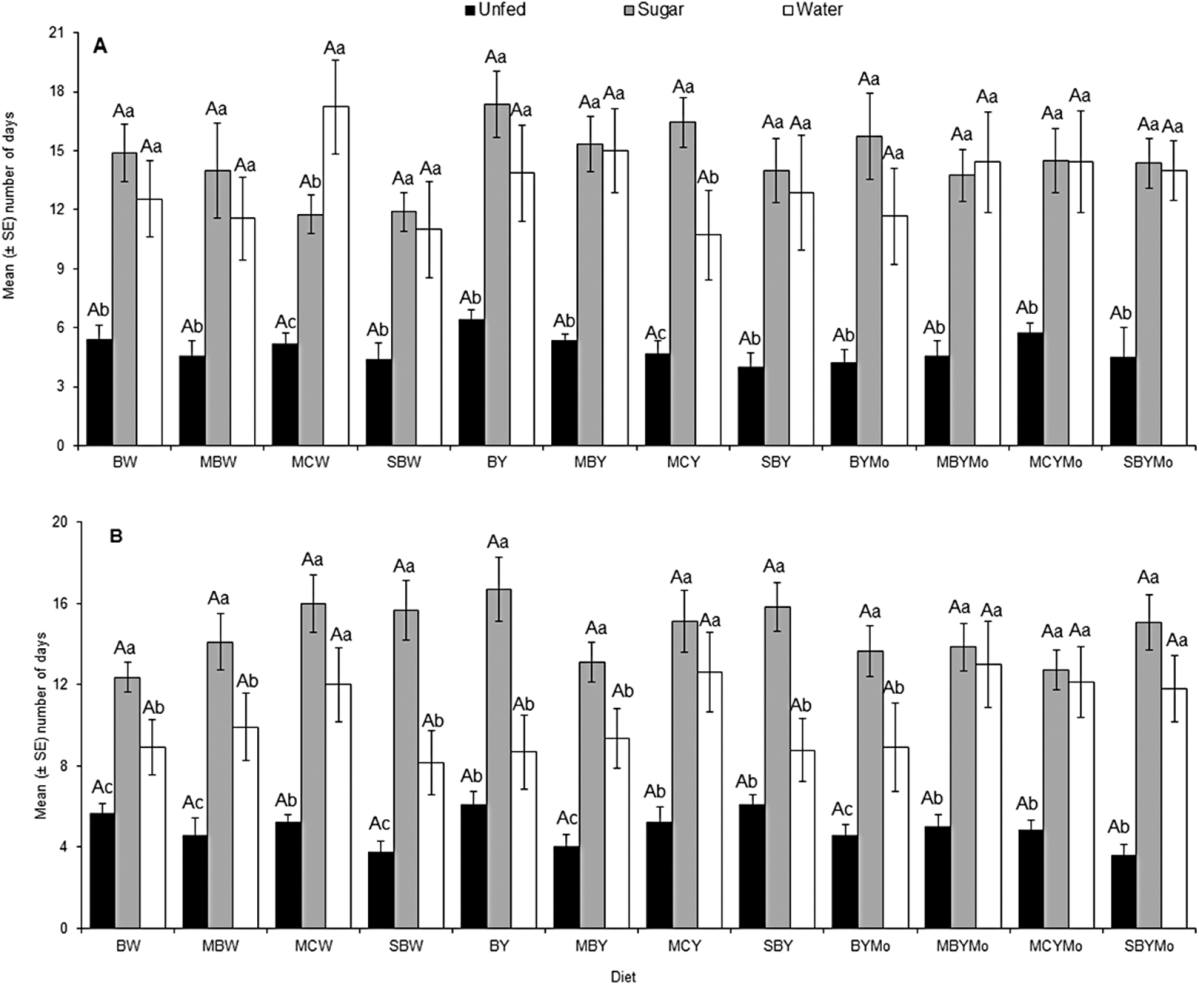
Longevity of adult male (A) and female (B) *H. illucens* fed on different diets as larvae and provided with sugar solution or water or remaining unfed as adults. Means (±SE) followed by different upper-case letters are significantly different among diets (*P* < 0.05, HSD). Means followed by different lower-case letter are significantly different among unfed, sugar-fed, and water-fed flies for each diet (*P* < 0.05, HSD). BW, Barley/water; MBW, Malt/Barley/water; MCW, Malt/Corn-starch/water; SBW, Sorghum/Barley/water; BY, Barley/brewer’s yeast; MBY, Malt/Barley/brewer’s yeast; MCY, Malt/Corn-starch/brewer’s yeast; SBY, Sorghum/Barley/brewer’s yeast; BYMo, Barley/brewer’s yeast/Molasses; MBYMo, Malt/Barley/brewer’s yeast/Molasses; MCYMo, Malt/Corn-starch/brewer’s yeast/Molasses and SBYMo, Sorghum/Barley/Molasses.

There was a significant interaction between adult food and sex of *H. illucens* (*F* = 5.99; d*f* = 2, 806; *P* = 0.004). However, no significant interaction was observed between larval diet and sex on adult fly longevity (*F* = 0.80; d*f* = 11, 806, *P* = 0.64). The longevity of both starved (unfed) male and female *H. illucens* was significantly lower (*F* = 208.79; d*f* = 2, 806; *P* < 0.001) compared to flies that were provided with sugar solution or water (Fig. 3).

### Effect of larval diet on wet weight of *H. illucens* life stages

**Figure 4:**
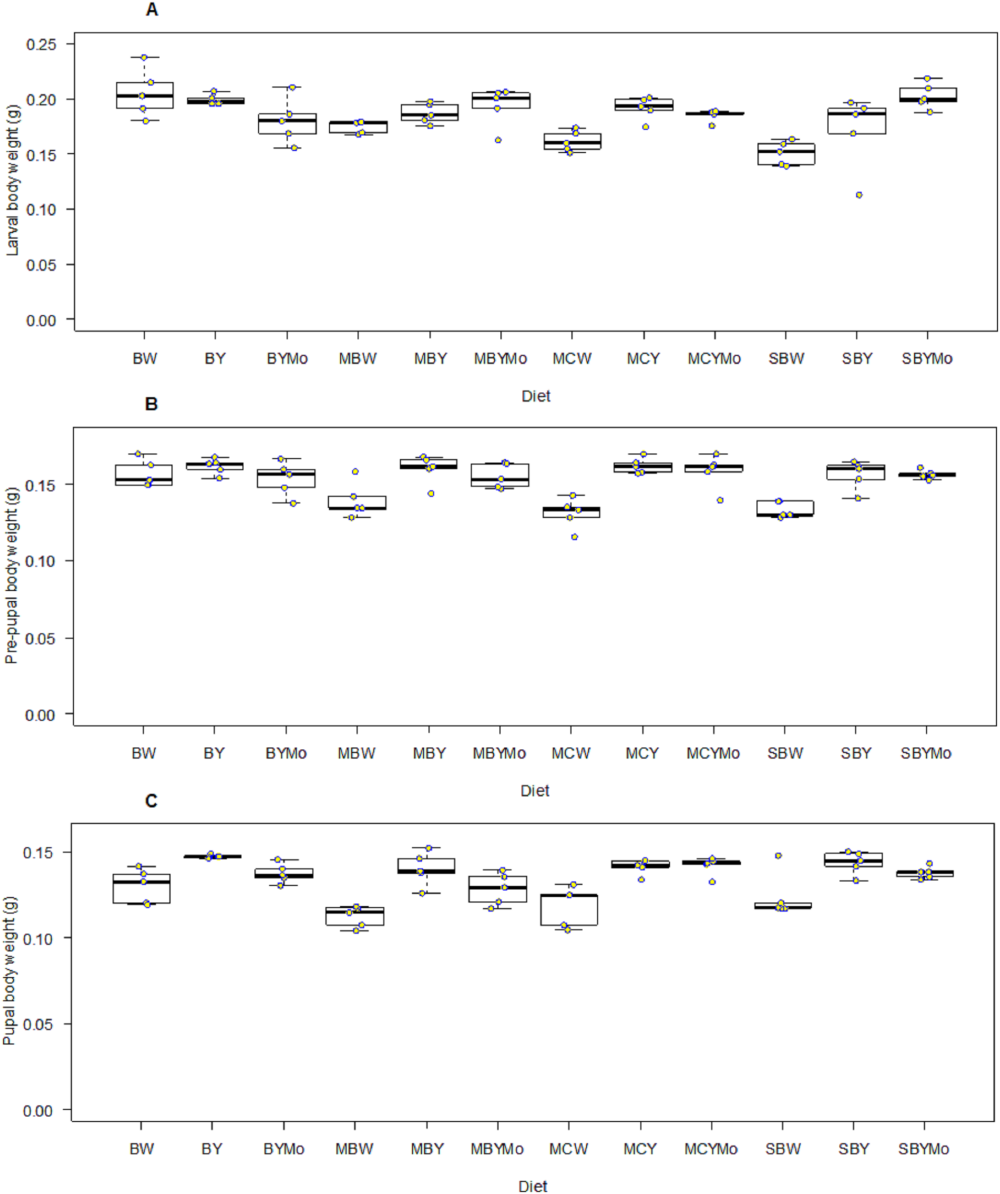
Boxplots showing wet weight (g) of larval (A), pre-pupal (B), and pupal (C) stages of *H. illucens* reared on different diet. The middle quartile or median (the line that divides the box into two parts) marks the midpoint of the data. The middle box (inter-quartile range) represents 50% of the data for each diet. BW, Barley/water; MBW, Malt/Barley/water; MCW, Malt/Corn-starch/water; SBW, Sorghum/Barley/water; BY, Barley/brewer’s yeast; MBY, Malt/Barley/brewer’s yeast; MCY, Malt/Corn-starch/brewer’s yeast; SBY, Sorghum/Barley/brewer’s yeast; BYMo, Barley/brewer’s yeast/Molasses; MBYMo, Malt/Barley/brewer’s yeast/Molasses; MCYMo, Malt/Corn-starch/brewer’s yeast/Molasses and SBYMo, Sorghum/Barley/Molasses.

**Figure 5:**
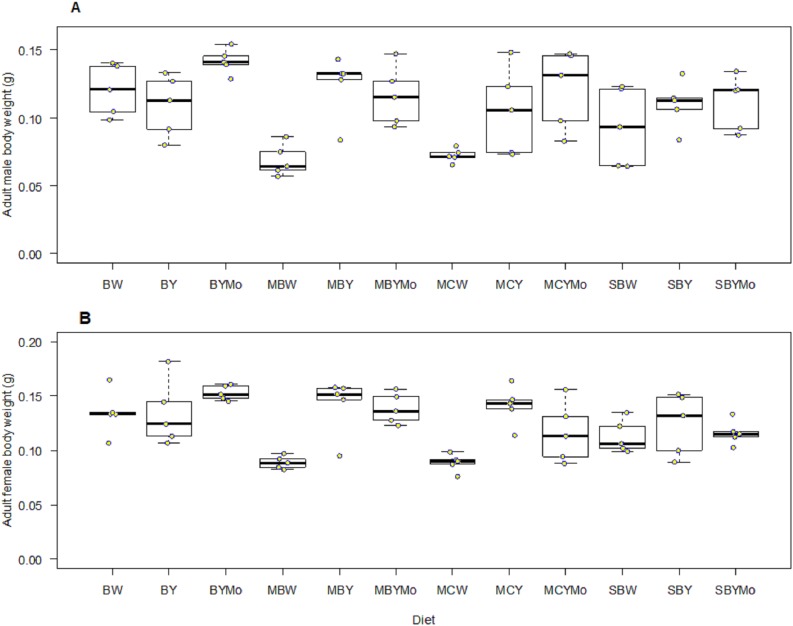
Boxplots showing wet weight (g) of adult male (A) and female (B) Black *H. illucens* reared on various diet. The middle quartile or median (the line that divides the box into two parts) marks the midpoint of the data. The middle box (inter-quartile range) represents 50% of the data for each diet. BW, Barley/water; MBW, Malt/Barley/water; MCW, Malt/Corn-starch/water; SBW, Sorghum/Barley/water; BY, Barley/brewer’s yeast; MBY, Malt/Barley/brewer’s yeast; MCY, Malt/Corn-starch/brewer’s yeast; SBY, Sorghum/Barley/brewer’s yeast; BYMo, Barley/brewer’s yeast/Molasses; MBYMo, Malt/Barley/brewer’s yeast/Molasses; MCYMo, Malt/Corn-starch/brewer’s yeast/Molasses and SBYMo, Sorghum/Barley/Molasses.

Fifth instar larval weight of *H. illucens* was significantly different (*F* = 5.46; d*f* = 11, 48; *P* < 0.001) among diets tested. Larval diet significantly affected weight of pre-pupa (*F* = 8.004; d*f* = 11, 48; *P* < 0.001), pupa (*F* = 9.08; d*f* = 11, 48; *P* < 0.001), adult male (*F* = 39.40; d*f* = 1, 96; *P* < 0.001), and female (*F* = 89.40; d*f* = 1, 96; *P* < 0.001) (Figs. 4 and 5). Larvae fed on non-supplemented diets weighed significantly (*F* = 106.3; d*f* = 1, 57; *P* < 0.001) less than those fed on diets supplemented with brewers’ yeast (Table 5). The weight of larvae fed on diets supplemented with brewer’s yeast or molasses/brewers’ yeast were not significantly different, whereas weight of pre-pupae differed significantly between non-supplemented diets and diets supplemented with either brewer’s yeast or molasses/brewers’ yeast (Table 5).

**Table 5:** Comparison of mean wet weight for different life stages of *H. illucens* between treatment groups using orthogonal contrasts.

Contrast	Larval weight	Pre-pupal	Pupal	Male	Female
	d*f* = 1, 48	d*f* = 1, 48	d*f* = 1, 48	d*f* = 1, 48	d*f* = 1, 48
Non-supplemented vs. Yeast/Molasses + yeast-supplemented diets	*F* = 12.80, *P* = **0.0008**	*F* =56.19, *P* < **0.0001**	*F* = 69.92, *P* < **0.0001**	*F* = 23.33, *P* < **0.0001**	*F* = 24.88, *P* < **0.0001**
Non-supplemented vs. Yeast only	*F* = 7.78, *P* = 0.07	*F* = 52.33, *P* < **0.0001**	*F* = 72.92, *P* < **0.0001**	*F* = 11.66, *P* = **0.013**	*F* = 21.76, *P* < **0.0001**
Yeast vs. Molasses/yeast	*F* = 0.38, *P* = 1.0	*F* = 2.20, *P* = 0.71	*F* = 6.73, *P* = 0.10	*F* = 2.36, *P* = 0.68	*F* = 0.48, *P* = 1.0
Barley vs. Corn-starch	*F* = 7.07, *P* = 0.09	*F* = 5.03, *P* = 0.23	*F* = 2.37, *P* = 0.67	*F* = 9.47, *P* = **0.038**	*F* = 12.11, *P* = **0.012**
Barley vs. Sorghum	*F* = 11.83, *P* = **0.013**	*F* = 7.77, *P* = 0.07	*F* = 1.12, *P* = 0.94	*F* = 5.75, *P* = 0.17	*F* = 9.99, *P* = **0.022**

**Notes:**

*P*-values in bold indicate significant difference.

Non-supplemented diets = BW, MBW, MCW, and SBW; Yeast/Molasses + yeast supplemented diets = BY, MBY, MCY, SBY, BYMo, MBYMo, MCYMo, and SBYMo; Yeast-based diets = BY, MBY, MCY, and SBY; Molasses/yeast diets = BYMo, MBYMo, MCYMo, and SBYMo; Barley = BW, BY, and BYMo; Corn-starch-based diets = MCW, MCY, and MCYMo; Sorghum-based diets = SBW, SBY, and SBYMo.

## Discussion

This study provides insight into the effects of mixing different waste types on *H. illucens* growth performance. We observed a shorter larval developmental duration in *H. illucens* reared on the 12 diet types compared to that documented in the literature (Cammack & Tomberlin, 2017; Oonincx et al., 2015; Nguyen, Tomberlin & VanLaerhoven, 2013; Myers et al., 2008). The larval developmental time in our study was 5–25 days shorter (17–21 days) than recorded in the studies mentioned above (21–46 days), a record similar to that reported by Barragán-Fonseca (2018). Differences in developmental time between studies may have been due to variation in the quantity and/or quality of the larval diet. A reduction in larval food supply could delay *H. illucens* larval development up to 4 months (Furman, Young & Catts, 1959). Other factors that affect larval development include larval density, larval feeding rate, and pH of the feeding medium (Barragán-Fonseca, 2018; Paz, Carrejo & Rodríguez, 2015; Morrison & King, 1977) as well as the physical texture of the feeding medium (Gobbi, Sánchez & Santos, 2013).

Pre-pupal recovery, pupal recovery, and adult emergence of *H. illucens* reared on diets supplemented with brewers’ yeast or molasses/brewery yeast compared favorably with, and sometimes exceeded, those obtained on the non-supplemented BSG diets. Percentage pupal recovery obtained for *H. illucens* was well within the range reported by several authors on a variety of rearing substrates (Oonincx et al., 2015; Nguyen, Tomberlin & VanLaerhoven, 2013; Myers et al., 2008). Percentage emergence of adults for the different diet types in our study was high, which agrees with previous studies (Cammack & Tomberlin, 2017; Tomberlin, Sheppard & Joyce, 2002). Considerably variable patterns have been reported for other dipterans like *Bactrocera dorsalis* (Hendel) and *Zeugodacus cucurbitae* (Coquillett) (Diptera: Tephritidae) that successfully completed development in diets containing the local waste stream, brewers’ yeast (Chang, Caceres & Ekesi, 2007; Chang, Caceres & Jang, 2004).

In our study, supplementation of BSGs with either brewers’ yeast or molasses/brewers’ yeast outperformed the spent grain diets mixed with water only in terms of increased larval, pre-pupal, pupal, and adult weight. The weight measurements of the different life stages observed in our study were comparable to those reported in previous research (Nguyen, Tomberlin & VanLaerhoven, 2013, 2015; Tomberlin, Adler & Myers, 2009; Tomberlin, Sheppard & Joyce, 2002). These studies report means of larval weight ranging from 0.11 to 0.23 g, pre-pupal weight of 0.065–0.22 g and adult weight of 0.044–0.111g, which are all similar to our findings. The stage-specific weight increase observed in our study has been viewed as a useful quality control criterion in insect mass-rearing since it is correlated with mating success. This result may be useful in the management of waste in a traditional brewing system which often generates very little (if any) value and may have negative impacts if the brewery must pay to get rid of waste water and spent grains. Adult flies from heavy pupae experience higher mating success than those from lower-weight pupae (Churchill-Stanland et al., 1986). Large flies exhibit greater flight ability than small flies (Sharp, Boller & Chambers, 1983). The difference in body weight observed in our study may be explained by the quality of diet and the resulting critical weight, one of the physiological factors that regulate variation in body size. The critical weight has been defined as the minimal mass at which further growth is not necessary for a normal time course to pupation (Davidowitz, D’Amico & Nijhout, 2003). Previous studies show that insects reared on low quality diets have low critical weight values (Davidowitz & Nijhout, 2004; Davidowitz, D’Amico & Nijhout, 2003).

Unlike the development of immature life stages, adult parameters (fecundity and adult longevity) were clearly affected by diet type on which the larvae were reared except for pre-oviposition duration. Previous studies have shown that in females, the nutritional quality of the diet (especially higher protein-based diets) ingested during the immature phase improves adult performance and affects ovarian development leading to higher fecundity rates (Cangussu & Zucoloto, 1993, 1995, 1997; Zucoloto & Fernandes-Da-Silva, 1997). Large protein-fed insect males are more likely to have their sperm stored in the females (Taylor & Yuval, 1999). Dietary effects on body size could be mediated through alterations in the quantity of nutrients stored as lipids and as proteins prior to pupariation (Nestel & Nemny-Lavy, 2008; Nestel, Nemny-Lavy & Chang, 2004). Nutrient composition of the brewers’ yeast (*Saccharomyces cerevisiae*) used is about 45% crude protein, (Raven & Walker, 1980) with an excellent lysine (amino acid) profile (Huige, 2006). BSGs contain 21–31% crude protein, and approximately 2,080 kcal/kg metabolizable energy on DM basis (Westendorf & Wohlt, 2002; National Research Council, 1994), but it is a poor source of other minerals (Hussain et al., 2010; Westendorf & Wohlt, 2002). Molasses is a source of readily available dietary energy (Van Niekerk, 1981), niacin, and pantothenic acid (Cleasby, 1963). We observed that diets supplemented with brewers’ yeast or molasses/brewers’ yeast resulted in slightly higher number of eggs produced than the non-supplemented diets, a trend similar to that observed by Barragán-Fonseca (2018) who recorded heavier *H. illucens* adults and higher egg production for diets with higher dietary protein contents.

Adult longevity of male and female flies was similar across diets when flies were starved. This implies that the nutritional quality of the larval diets appears to have had minimal effects on life span of the flies and significant impact on egg production (no eggs were laid). Water, unlike food, is essential for adult *H. illucens* to reproduce (Sheppard et al., 2002), which might explain why less vigorous and dehydrated adults were unable to lay eggs. In our experiment, adult longevity increased with 10% sugar solution or water supply as food in separate treatments. The longevity of male and female flies varied on each diet type when adult flies were subjected to water only or 10% sugar solution treatments. Although we did not evaluate the effects of protein and carbohydrate on adult *H. illucens* longevity, previous research has indicated that dietary protein and carbohydrate contents are important and affect both larval and adult performance of *H. illucens* (Barragán-Fonseca, 2018).

## Conclusion

The successful development of *H. illucens* on all 12 diet types clearly indicates the high nutrient quality of the breweries by-products, especially the protein/NE balance, which has been demonstrated to be optimal for maximum production (Clifford & Woodring, 1990; Woodring, Clifford & Beckman, 1979). Based on quality control parameters of *H. illucens reared on the combination of these agro-industrial waste streams, our values are* comparable to, and sometimes higher than, those *documented in literature on other organic wastes.* Thus, the study at hand confirms the application potential of the Black Soldier Fly in industrial solid waste management and the importance to investigate future large-scale mass rearing possibilities. The combination of waste treatment capacity together with generation of a valuable product, that is, a high-quality cheap alternative protein source for animal feeds, instead of discarding the waste into open plots, on streets or in rivers that attracts scavenging animals as well as disease spreading insects, makes the Black Soldier Fly technology a highly promising tool for waste management. The conversion of organic waste into high nutritional biomass has now opened new economic opportunities for municipalities and offers small entrepreneurs the possibility of income generation without high investment costs, and concurrently reduces the environmental impact of organic waste stream currently considered one of the most immediate and serious environmental problems confronting urban governments in low- and middle-income countries in Sub-Saharan Africa. Hence, composting by utilizing Black Soldier Fly larvae should be recommended in Kenya and other African countries as a sustainable method of dealing with organic municipal waste that embraces the concept of a circular economy. Being a financially more attractive option for municipal waste management, private sectors, with stronger focus in business opportunities and marketing approaches should be in the center of attention (Fluitman, 2000; Wang, Han & Li, 2008).

## Supplemental Information

10.7717/peerj.5885/supp-1 Supplemental Information 1 Raw data: adult longevity (female).

10.7717/peerj.5885/supp-2 Supplemental Information 2 Raw data: development time.

10.7717/peerj.5885/supp-3 Supplemental Information 3 Raw data: nutrient composition of experimental diets.

10.7717/peerj.5885/supp-4 Supplemental Information 4 Raw data: percent survival (larva, prepupa, pupa).

10.7717/peerj.5885/supp-5 Supplemental Information 5 Raw data: adult longevity (male).

10.7717/peerj.5885/supp-6 Supplemental Information 6 Raw data: number of eggs laid.

10.7717/peerj.5885/supp-7 Supplemental Information 7 Raw data: pre-oviposition time.

10.7717/peerj.5885/supp-8 Supplemental Information 8 Raw data: wet weight.

## Additional Information and Declarations

### Data Availability

The following information was supplied regarding data availability: The raw data are provided in the Supplemental Files.
